# Noncoding RNAs orchestrating the central dogma

**DOI:** 10.1016/j.jbc.2025.110933

**Published:** 2025-11-14

**Authors:** Sebastian Lozano-Villada, Sathyanarayanan V. Puthanveettil

**Affiliations:** Department of Neuroscience, The Herbert Wertheim UF Scripps Institute for Biomedical Innovation & Technology, Jupiter, Florida, USA

**Keywords:** central dogma, RNA function, non-coding RNA, RNA modifications, molecular biology, gene regulation, neurons

## Abstract

Once considered mere transcriptional noise, noncoding RNAs (ncRNAs) are now recognized as central players in cellular function—serving as scaffolds, catalysts, and regulators of gene expression. Their pervasive influence compelled a fundamental reevaluation of the central dogma of molecular biology, which traditionally emphasized the linear flow of genetic information from DNA to RNA to protein. In this review, we highlight critical roles played by ncRNAs and the outstanding challenges in decoding their mechanisms of action. We focus in particular on the emergent view that ncRNAs operate as components of highly integrated and dynamic regulatory networks. These RNA interactomes are governed by precise spatiotemporal constraints yet remain flexible enough to adapt to shifting cellular demands. Key questions remain about how such networks are prioritized, regulated, and dynamically reshaped in response to physiological signals. To contextualize these mechanisms, we spotlight the nervous system, a tissue enriched in regulatory RNAs, as a model for understanding how ncRNA-mediated regulation contributes to cellular specialization and plasticity. Unraveling the principles governing ncRNA interactions will reveal new dimensions of the genetic code, one that is not simply read but interpreted and orchestrated by RNA.

Francis Crick proposed two foundational concepts that shaped modern molecular biology: (1) the Sequence Hypothesis and (2) the Central Dogma. Together, these models described a unidirectional flow of genetic information from DNA to RNA to protein, with rare exceptions (1, 2). For decades, these ideas stood largely unchallenged, positioning DNA as the primary source of genetic information, proteins as the key effectors of cellular function, and RNA as an intermediary. However, advances in molecular biology have significantly expanded this framework, revealing RNA to be far more than a passive messenger. RNAs are now recognized as dynamic regulators in diverse cellular processes, often operating under stringent spatial and temporal constraints.

A pivotal shift occurred in 2003 with the completion of the Human Genome Project, which revealed that less than 2% of the human genome encodes proteins. This surprising finding catalyzed a surge of interest in the remaining vast noncoding regions, particularly in noncoding RNAs (ncRNAs)—including microRNAs (miRNAs), small-interfering RNAs (siRNAs), PIWI-interacting RNAs (piRNAs), and long-noncoding RNAs (lncRNAs). These transcripts have since been shown to orchestrate gene expression through intricate and often evolutionarily conserved mechanisms (3, 4, 5, 6, 7, 8).

Crucially, these findings have challenged the simplicity of the linear transcription–translation model, emphasizing instead that biological systems operate through complex, multi-layered networks of RNAs which seldom function in isolation. Rather, they engage in dynamic interaction networks with other RNAs, proteins, DNA, lipids, and carbohydrates. This interactome provides a lens through which to view RNA function, but it also raises fundamental questions: How are RNA interactions prioritized and regulated within the crowded cellular environment? To what extent are these interactions stochastic *versus* directed by structural, chemical, or spatial cues? And how do RNA modifications and localization patterns influence interaction dynamics across different cell types?

These questions become particularly relevant in the nervous system, where neurons exhibit high compartmentalization, longevity, and translational control. Neurons depend on precise spatial and temporal regulation of RNA metabolism—including synthesis, processing, transport, modification, storage, and degradation—to maintain circuit function and respond to synaptic stimuli. Disruptions in any of these steps can lead to disproportionate cellular dysfunction, often manifesting in neurodevelopmental or neurodegenerative disease. Understanding how neurons leverage the RNA interactome offers not only mechanistic insight into RNA biology but also a model for exploring broader principles of spatiotemporal gene regulation.

In this review, we first consider the unique attributes of neuronal RNA metabolism that make these cells especially vulnerable to perturbations in the RNA interactome. We then discuss five major challenges in decoding RNA function—diversity, structure, conservation, interaction complexity, and post-transcriptional regulation—and show how these features converge in neurons. Finally, we explore how mutations in noncoding RNAs influence susceptibility to neuropsychiatric disease, offering a deeper view into how RNA-centered mechanisms shape the nervous system.

## RNA metabolism in neurons

Neurons are specialized cells that are post-mitotic, long-lived, and remarkably compartmentalized. Their unique morphology, with axons and dendrites extending from millimeters to over a meter away from the soma, poses extraordinary challenges for gene regulation. Large neurons, such as motor neurons and Purkinje cells, are particularly vulnerable to disruptions in RNA metabolism due to the long distances over which RNA molecules must be trafficked and locally regulated (9, 10). This architecture demands spatially and temporally precise gene expression that is both rapid and plastic, a requirement in which RNA molecules, particularly noncoding RNAs (ncRNAs), play a central role.

RNA’s intrinsic biophysical properties, including rapid synthesis, conformational flexibility, and context-dependent decay, make it an ideal regulatory molecule for neurons, where responses must be both localized and temporally fine-tuned to communicate with potentially thousands of other cells (11). RNA secondary structure enables dynamic engagement with distinct sets of RNA-binding proteins (RBPs) and miRNAs, allowing for adaptive regulatory outcomes (12, 13). This structural versatility underpins diverse functions, from riboswitch-like control elements to lncRNAs that serve as scaffolds within RNP granules (14, 15). In such a system, local translation of pre-existing mRNAs is faster and more efficient than relying on soma-to-synapse transport of newly synthesized proteins. This feature favors RNA-based regulation, which can often be modulated by reversible post-transcriptional modifications, such as N6-methyladenosine (m^6^A), to influence RNA stability, translation, and subcellular localization (16). Disruptions in RNA granule trafficking or RNP remodeling underlie pathogenesis in ALS and FTD, particularly in models involving TDP-43 or FUS mutations (17).

Recent transcriptomic and proteomic studies have revealed that neurons are enriched for metabolic pathways tailored to support their specialized RNA requirements. For example, glycine N-methyltransferase (GNMT), an enzyme typically confined to the liver, is expressed in hippocampal neurons, where it regulates the S-adenosylmethionine/S-adenosylhomocysteine (SAM/SAH) ratio and thereby influences cellular methylation capacity, including RNA methylation (18, 19). GNMT knockout mice exhibit impaired neurogenesis in the dentate gyrus and subventricular zone, emphasizing the role of methylation in neural development. Similarly, deficiencies in methylenetetrahydrofolate reductase (MTHFR) have been linked to neural tube defects and impaired neurogenesis, underscoring the importance of methionine-cycle enzymes in neuronal function (20, 21).

Moreover, neurons exhibit regionally compartmentalized metabolic zones, with mitochondria positioned at active synapses and translational machinery distributed to meet local demands (22, 23). Reductions in mitochondrial transport flux and velocities along axons, resulting from disrupted motor and adaptor protein dynamics, leads to local ATP depletion and synaptic dysfunction (24). Notably, RNA granules colocalize with mitochondria, positioning them to integrate energy and calcium cues critical for localized translation (25, 26, 27). These interdependencies highlight the spatial and metabolic susceptibilities of neurons to disruptions in RNA regulation, susceptibilities far more pronounced than in non-neuronal cells.

As a byproduct of synaptic activity, neurons produce large amounts of oxidized RNA that is often not cleared immediately (28). To preserve fidelity, neurons deploy highly specialized RNA quality control mechanisms, including compartment-specific degradation *via* nuclear exosomes and cytoplasmic nonsense-mediated decay (NMD) (29). In fact, studies in *C*. *elegans* and *D*. *melanogaster* have shown that NMD activity decreases with age in the muscle, hypodermis, and intestines, but not in neurons (30, 31). Disruption of these pathways, for example, by mutations in UPF3B or EXOSC3, results in neurological phenotypes such as intellectual disability and pontocerebellar hypoplasia (32, 33). These findings underscore the idea that neurons have a reduced tolerance for RNA processing errors and transcriptional noise.

The functional diversity and abundance of ncRNAs in the nervous system further amplify the consequences of their dysregulation (34, 35). ncRNAs not only regulate gene expression but also participate in the formation of phase-separated condensates, chromatin organization, and the spatial control of protein synthesis (36). These roles are especially critical in synaptic plasticity and activity-dependent transcription. For example, nuclear-enriched lncRNAs such as MALAT1 and Gm12371 regulate synapse density and excitability by modulating synaptic protein expression (37, 38, 39). Dysregulation of NEAT1 perturbs paraspeckle dynamics and has been linked to frontotemporal dementia and depression (40, 41). Likewise, neuronal miRNAs such as miR-124 and miR-132 govern dendritic growth, spine morphogenesis, and memory consolidation, with disruptions contributing to neurodevelopmental and cognitive disorders (42, 43).

Thus, neurons present unique opportunities and challenges for a deep examination of the central dogma in our current RNA-centric biology. Their architecture, metabolic compartmentalization, and reliance on localized, rapid, and precisely regulated RNA processing demand molecular strategies not seen in other cell types. This complexity renders the nervous system both an exemplary environment for RNA biology and a sensitive indicator of its dysregulation.

## The emerging complexity of the RNA interactome

**Figure 1:**
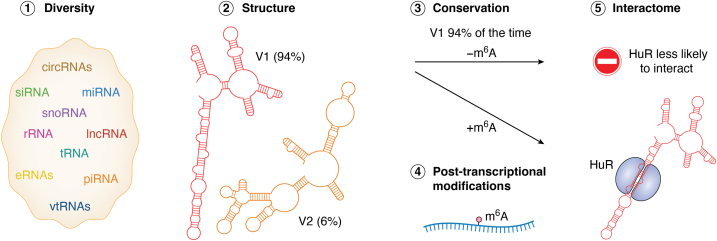
**Five Key Challenges in Understanding Noncoding RNA-Mediated Mechanisms**. This figure highlights five major challenges in RNA biology—diversity, structure, conservation, interactions, and post-transcriptional modifications—using a conformationally variable region of lncRNA MALAT1 (nucleotides 5088–5349) (44) (1) The diversity of ncRNAs (*e*.*g*. miRNAs, lncRNAs, siRNAs, snoRNAs, piRNAs) and functionally distinct isoforms within a class adds substantial complexity. (2) Structural variability in the selected region of MALAT1 reveals two alternative conformations (Version 1 (V1) and V2), each with distinct binding motifs, exemplifying the challenge of predicting RNA structure-function relationships. (3) While the primary sequence of many lncRNAs is poorly conserved, their secondary structures can be conserved across species and exist predominantly as one isoform, as seen in V1. (4) Post-transcriptional modifications like N6-methyladenosine (m^6^A) can enhance transcript stability, as seen in this region of MALAT1 (45). (5) The same conserved structure may engage in diverse interactions depending on cellular context; in this example, m^6^A preferentially promotes HuR binding (46).

The discovery of extensive ncRNA populations and the regulatory properties of messenger RNAs (mRNAs) have collectively reshaped our understanding of gene regulation. ncRNAs play versatile roles beyond their coding potential, including direct interactions with mRNAs, scaffolding and transporting biomolecules, catalysis, and compartmentalization of cellular processes through bimolecular condensates. Together, these diverse functions integrate into a remarkably complex “RNA Interactome”; however, the coordination of these seemingly independent roles remains difficult to define. In neurons, with specialized cellular compartments and high demands for plasticity, ncRNAs play key roles in driving neural circuit function. Certain lncRNAs and miRNAs are enriched in key brain areas, modulating gene expression through local and long-range mechanisms, and often in direct response to neuronal activity. This coordination is essential for synaptic remodeling, memory formation, and overall circuit homeostasis. Below, we explore five key regulatory challenges that shape the RNA interactome (Fig. 1), and discuss emerging technologies being used to resolve these challenges.

### Diversity

A central challenge in dissecting the RNA interactome lies in the sheer diversity of RNA species. ncRNAs encompass a wide array of molecules, including long non-coding RNAs (lncRNAs), small interfering RNAs (siRNAs), microRNAs (miRNAs), circular RNAs (circRNAs), and ribosomal RNAs (rRNAs), among others. These classes are based predominantly on length and structure, but also on varying functions, such as chromatin modification or modulation of mRNA stability. However, the challenge lies further than cataloging these ncRNAs and focuses rather on understanding how their functions interconnect within broader regulatory networks, especially in complex tissues like the brain. Here, the diversity is not only with respect to the classes of ncRNAs, but also to their location.

ncRNAs exhibit strikingly distinct expression profiles in the nervous system, with approximately 40% of lncRNAs reported to be specifically enriched in the brain (36, 47, 48). One prominent lncRNA, MALAT1, is abundant in neurons and shapes synaptic function and plasticity by regulating alternative splicing and protein interactions (37). Beyond global enrichment in the brain, unique ncRNA localization patterns emerge in different brain regions. For instance, the hippocampus, which is key to memory formation, exhibits a lncRNA signature distinct from the amygdala or prefrontal cortex (49, 50). Other ncRNAs, including miRNAs like miR-134, show similar nervous system specificity. miR-134 is localized in the hippocampus and inhibits an mRNA encoding protein kinase Limk1, which regulates spine development (51). The miR-183/96/182 cluster is also strongly expressed in neurons and is implicated in processes such as sensory processing and neuronal development (52).

While these ncRNAs are generally expressed differentially throughout the nervous system, their localization within individual neurons is critical (53). The localization of RNA within subcellular compartments allows it to respond rapidly to stimuli, modulate local protein synthesis, and alter gene expression necessary for memory formation. Nuclear retained ncRNAs, especially lncRNAs, are known to modulate chromatin structure and accessibility. Transcripts such as NEAT1 and MALAT1, which are more abundant in neurons than in other tissues, can form phase-separated condensates (*e*.*g*. paraspeckles and nuclear speckles) that modulate the expression of genes vital for synaptic function (54, 55, 56). By integrating signals arriving from dendritic and synaptic compartments, these nuclear-retained ncRNAs help tune long-term transcriptional responses in neurons.

This complexity is compounded by the fact that these RNAs often interact with the same protein partners, share processing machinery, or even antagonize each other’s functions. For example, miRNAs and siRNAs both rely on the RISC complex but differ in their biogenesis and target specificity (57). Similarly, circRNAs derived from neuronal genes can sequester miRNAs or RBPs, thereby competing with linear transcripts for interaction partners (58). In some cases, RNAs transcribed from the same genomic locus can exert opposing effects; for example, antisense transcripts may either enhance or suppress the expression of their corresponding sense mRNAs.

Given this diversity, traditional methods for RNA annotation and quantification remain insufficient. While short-read RNA-seq has revolutionized transcript discovery, it often fragments structured RNAs, misses circular transcripts, and underrepresents short regulatory RNAs like tRFs (tRNA-derived fragments). These limitations are especially pronounced in neurons, where a significant portion of the regulatory RNA pool is localized, low-abundance, and isoform-rich. Therefore, single-cell long-read technologies, like PacBio Iso-Seq or Oxford Nanopore, have become increasingly useful to capture full-length isoforms in a single pass (59).

Equally important is the need for absolute quantification. Neurons frequently rely on stoichiometric competition between RNAs, such as miRNA sponges or decoy lncRNAs, and knowing the relative abundance of these transcripts is essential. Techniques like spike-in calibrated qPCR and digital droplet PCR offer valuable complementary data that sequencing alone cannot provide (60, 61, 62). These measurements can be used to prioritize candidates based not only on expression, but on whether they are present at sufficient levels to functionally impact their targets.

The next step lies in translating these assays into comprehensive databases that integrate large and small RNA populations from diverse tissues, developmental stages, and pathological contexts. While current databases (*e*.*g*. ENCODE, GTEx) have indeed expanded coverage of ncRNAs, a unified view remains elusive. Over 100,000 lncRNAs have been recorded in humans, yet most remain uncharacterized, and many oftentimes go undetected due to transient expression states (8, 63).

### Structure

Beyond sequence diversity, ncRNAs adopt various secondary and tertiary structures that dictate their regulatory roles (Fig. 1). Unlike proteins, whose higher-order structure is often stabilized by extensive hydrophobic cores, RNAs can shift among multiple conformations with relatively small energetic differences. Indeed, the roles of riboswitches and biomolecular condensates hinge upon the specific folds and multivalent interaction regions that transcripts can adopt. In many instances, local secondary elements interact synergistically, shaping higher-order structures whose features determine the assembly and regulation of crucial complexes with other macromolecules. In neurons, where synaptic signals can propagate within milliseconds and local protein expression must be tightly synchronized with neuronal activity, the plasticity of RNA structure may serve as a central organizing feature of spatiotemporal processes.

**Figure 2:**
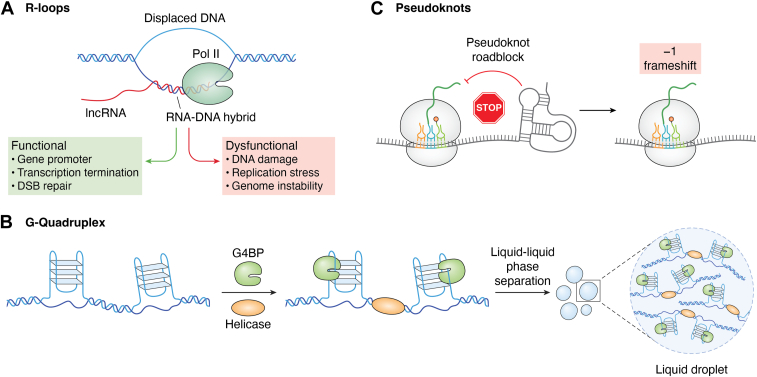
**Structural Features of RNA and Their Roles in Gene Regulation**. *A*, R-loops form when a single-stranded RNA molecule, in this case a lncRNA, displaces the non-template strand of double-stranded DNA and forms an RNA-DNA hybrid with the template strand. These structures can have both functional and dysfunctional roles, such as aiding in double-stranded break repair or adding instability to the genome and causing DNA damage (64, 65). *B*, pseudoknots arise from intramolecular base pairing with the same RNA strand and commonly act as translational roadblock, triggering ribosomal frameshifts and altering translational output (66). *C*, G-quadruplexes are guanine-rich RNA secondary structures that promote phase separation into biomolecular condensates through interactions with RNA-binding proteins and helicases (67). LLPS: liquid-liquid phase separation; G4BP: G-quadruplex-binding protein.

Among the most functionally significant RNA structures are R-loops, G-quadruplexes, and pseudoknots, each of which plays a distinct role in gene regulation (Fig. 2). R-loops are three-stranded hybrid structures formed when an RNA transcript hybridizes with its DNA template (68, 69) (Fig. 2). Today, these structures are increasingly recognized as regulators of transcription and chromatin state and can facilitate gene activation by preventing nucleosome reassembly (70, 71). Interestingly, excessive accumulation of R-loops can lead to genomic instability by causing replication fork stalling and even DNA damage, raising the question of how cells balance the levels of these structures in specific contexts (72, 73). R-loop stability is influenced by certain ncRNAs, like lncRNA Lnc530, which localizes on R-loops and scaffolds various proteins like DDX5 and TDP-43 to stabilize these structures (74). In neurons, high levels of transcription also increase susceptibility to the formation of R-loops, which can heighten the risk of DNA damage (75).

G-quadruplexes are secondary structures formed in guanine-rich sequences, where four guanine bases form a stable structure through Hoogsteen hydrogen bonding (76) (Fig. 2). Interestingly, these complex structures are enriched ∼3-fold near splice junctions, where they have been shown to modulate alternative splicing, potentially altering the availability of binding sites for translation initiation factors and RNA-binding proteins (RBPs) (77). Additionally, G-quadruplexes play roles in telomere maintenance and DNA replication by serving as capping structures and promoting replication fork stalling, respectively (78, 79). Notably, G-quadruplexes also promote liquid-liquid phase separation (LLPS) by enhancing RNA-protein interactions within these membrane-less organelles (80, 81) (Fig. 2). This ability to drive phase separation through multivalent interactions also makes them critical components of synaptic granules and nuclear bodies (82).

Pseudoknots are another important RNA structure that plays roles in translation initiation and viral replication (83, 84, 85) (Fig. 2). Pseudoknots are known for their ability to interact with ribosomes and translation initiation factors to modulate protein synthesis (66, 86, 87). In viruses, pseudoknots are involved in frameshift mechanisms during translation, which leads to the production of alternative protein products from the same mRNA transcript (88) (Fig. 2). It is plausible that cytoplasmic RNAs use similar mechanisms to those seen in viruses for endogenous regulatory control.

While R-loops, G-quadruplexes, and pseudoknots illustrate the diversity of RNA structural conformations, additional layers compound this complexity. Riboswitches are active RNA structures that pair an aptamer domain that senses small molecules (*e*.*g*. amino acids, cofactors) to an expression platform that toggles between alternate folds to govern transcription termination or translation initiation (89, 90, 91). From a broader perspective, these motifs foreshadow a theme common to many regulatory RNAs: functional specificity often depends more on shape than sequence, enabling certain structures to recur throughout evolution.

Scaffolding capacity likewise emerges from a RNA’s ability to fold into discrete subdomains that simultaneously accommodate multiple binding partners. Long non-coding RNAs, in particular, have gained prominence for their scaffolding roles (92). RNAs such as XIST, MALAT1, and NEAT1 harbor distinct domains that adopt discrete folds to spatially arrange chromatin modifiers, splicing regulators, or transcription factors (93, 94, 95). Each domain can selectively recruit proteins based on secondary motifs or base-pairing potential, allowing a single lncRNA to organize complex condensates like paraspeckles or nuclear speckles (92, 96, 97). Changes in RNA structure—whether driven by neuronal activity or post-transcriptional modifications—can modulate condensate organization by altering RNA–RBP interactions and RNA flexibility. These structural alterations, in turn, influence the stoichiometry of condensate components and the spatial volume occupied by RNAs within them. (98). In neurons, where spatial organization is tightly linked to synaptic plasticity, these structural shifts may directly influence signal transduction or transcriptional feedback loops.

CircRNAs, enriched in neural tissue, exhibit remarkable scaffolding or “sponging” behavior (99). The circRNA ciRS-7, for instance, contains numerous binding sites for miR-7, effectively locking away the miRNA from its typical mRNA targets (58, 100). CircRNAs accumulate in synaptic fractions and may contribute to local translation through structure-dependent binding, a feature that warrants deeper exploration in the context of dendritic and axonal function (101, 102). A step further than scaffolding, biomolecular condensates serve as the bridge between structural regulation and cellular organization. These dynamic, membrane-less compartments (such as stress granules, P-bodies, nuclear speckles, and paraspeckles) focus biochemical reactions by concentrating select proteins and RNAs. RNAs often serve as pivotal organizers in these condensates, providing multiple low-affinity binding sites that collectively drive phase separation (103, 104).

The role of ncRNAs in cellular spatial compartment organization is especially crucial in the nucleus (105). For example, the lncRNA NEAT1 is essential to paraspeckle assembly, harboring regions that fold to expose motifs recognized by SFPQ, PSPC1, and other proteins (96). In nuclear speckles, MALAT1 accumulates splicing regulators, though it may not be strictly essential for speckle formation (92, 97, 106). A key principle is that these RNAs engage in multiple interactions simultaneously: certain structural elements tether them to a nuclear subdomain, while others bind RBPs that can themselves contain intrinsically disordered regions conducive to phase separation. This phenomenon is especially prominent in neurons, where RNA-containing granules orchestrate the local response to synaptic activity.

Currently, techniques have focused on cataloging stable RNA folds under purified conditions, yet the next step in overcoming the structure challenge is to capture conformational states inside living cells. *In vitro* approaches like X-ray crystallography and NMR typically cannot accommodate large, flexible RNAs or the dynamic interplay between RNA and binding partners. Instead, the field has embraced *in vivo* chemical probing tools, such as SHAPE or DMS footprinting, coupled with high-throughput sequencing (107, 108). Compartment-resolved icSHAPE now reveals how the same transcript adopts distinct folds on chromatin, in the nucleoplasm, or in the cytoplasm, linking structure to transcription, translation, and decay (109). An exciting frontier involves merging immunoprecipitation (IP) with chemical probing (*e*.*g*. IP-SHAPE-seq, IP-DMS-seq) to elucidate how a given RBP’s binding reshapes local RNA structure (110, 111, 112). In neurons, such tools could clarify how activity-dependent RBPs induce structural transitions that drive plasticity or memory encoding. For example, neuronal stimuli could trigger conformational changes in dendritic lncRNAs, enabling selective recruitment of translational machinery or initiation of condensate formation.

Future directions in the structure challenge must prioritize context-dependent structure mapping in intact tissues, especially in the brain. Single-cell structure probing would allow direct linkage of structure to function across neuronal subtypes. New chemical modifiers with faster kinetics or transient reactivity could reveal folding dynamics over seconds rather than minutes. Additional chemical tools, including advanced SHAPE or DMS variants sensitive to partial modifications, hold promise for capturing transitional structures that flicker in and out of existence (113, 114). Ultimately, a deeper understanding of how RNAs fold, refold, and scaffold large complexes stands to illuminate a new dimension of cellular regulation, bridging biochemical specificity and large-scale spatial organization.

### Conservation

Despite growing evidence of the functional relevance of various RNA structures, a major challenge in RNA biology lies in understanding the conservation of these structural elements across species. In protein-coding genes, sequence-level conservation often directly reflects selective pressure to maintain amino acid residues essential for protein function. By contrast, many lncRNAs, circRNAs, and structured mRNAs show low primary-sequence conservation. However, disrupting even a few nucleotides in certain functional structural motifs can abolish their activity, suggesting that structural rather than sequence-level features are often under intense evolutionary constraint (115, 116). This mismatch between conventional definitions of sequence homology and functional conservation presents an intriguing question: Are these conserved structural domains sufficient to execute function when sequence conservation is absent?

Recent studies suggest that certain RNA structures are indeed essential for the regulatory functions of lncRNAs. The conserved pseduknots in lncRNA MEG3, for example, are necessary for interactions with specific RNA-binding proteins that are central to the lncRNA’s role in gene silencing for tumor suppression and p53 activity regulation (117). Riboswitch aptamer domains for cofactors like thiamine pyrophosphate (TPP) also show high structural conservation even in evolutionarily distant organisms, verifying that the 3D fold is the main driver of selective pressure (118). In biomolecular condensates, lncRNA NEAT1 homologs in various mammals appear to retain functional repeats for SFPQ/NONO binding, even if flanking regions differ significantly (96). Similarly, TERRA transcripts incorporate repetitive telomeric sequences that remain functionally important across eukaryotes, from yeast to humans (119).

Interestingly, miRNAs provide a contrasting example of conservation in RNA biology. Unlike lncRNAs, miRNAs are typically highly conserved at the primary sequence level, likely due to being only 20 to 24 nucleotides long. However, despite remarkable sequence conservation, miRNA functionality is complicated by their ability to regulate numerous targets across diverse pathways. For example, miR-21 targets PDCD4, which is pro-apoptotic and promotes cell death, while also targeting BCL2 which is anti-apoptotic and helps cancer cells evade death (120). In contradictory cases like this situation, studying miRNA function becomes problematic.

Exciting approaches, such as the phylo-informed RNA folding framework, can detect conserved folding patterns in the absence of precisely conserved sequences among different lineages. For example, detecting a G-quadruplex motif that emerges in all mammalian orthologs of a novel lncRNA, even if the guanine repeats shift in spacing or partial base composition. In neurons, evolutionary rewiring may be key to adapting conserved functional roles to diverse developmental and behavioral contexts. One lineage might use a G-quadruplex to stall ribosomes during synaptic scaling, while another uses a pseudoknot to recruit similar effectors. These mechanisms reflect convergent evolution at the level of fold, rather than code. Just as enhancers evolve rapidly while maintaining core outputs, regulatory RNAs may tolerate high sequence drift while preserving interaction. To decipher these mechanisms, Infernal (INFERence of RNA Alignment) and Rfam are able to build and store covariance models for RNAs that capture both sequence and structural conservation (121, 122). Once this covariance model is built, it can be used to search other genomes and identify RNAs that differ in sequence but share conserved structure.

Finally, it is worth noting that evolutionary change does not always imply functional loss. In some cases, structural motifs can be replaced by others that serve analogous roles, an example of “structural rewiring.” Indeed, some lineages might develop alternative hairpin or quadruplex arrangements that approximate the same net effect, such as binding the same RBP or controlling the same splicing event. This plasticity complicates simple attempts to classify RNAs as either “conserved” or “nonconserved,” reinforcing the importance of careful experimental validation. Nevertheless, when a structural element persists across large evolutionary distances, it frequently signifies a robust functional anchor in the transcript’s biology.

### Interactome

The complexity of the RNA interactome is determined not only by RNA diversity and structure, but also by the vast interaction possibilities with various macromolecules (Fig. 1). Dynamic rearrangements of RNA structure and subcellular localization can remodel an RNA’s binding partners at any given moment, leading to cascades of changes in gene expression. Moreover, the same RNA often functions in distinct contexts, serving as a scaffold for ribonucleoprotein (RNP) complexes in one cell state and as a substrate for RNA modifications in another This fluidity is particularly pronounced in neurons, where synaptic activity and intracellular compartmentalization constantly reshape the composition and function of RNA-bound assemblies.

Neurons rely on activity-dependent control of RNA localization to implement rapid, localized changes in protein expression and signal transduction. While much focus has been on mRNAs encoding synaptic proteins, it is now well understood that ncRNAs play pivotal roles in shaping synaptic plasticity. These ncRNAs, especially lncRNAs, translocate to precise subcellular locations in response to neuronal activity. Once localized, they may coordinate translation and kinase pathways essential for sustained changes in synaptic strength.

For example, lncRNA ADEPTR is upregulated in response to synaptic activity and subsequently transported to dendrites dependent on cAMP/PKA signaling (123). ADEPTR interacts with actin-scaffolding regulators, like AnkB and Sptn1, and mediates their localization to dendrites. Loss of ADEPTR impairs changes in dendritic spine morphology, which are essential for memory consolidation. Similarly, lncRNA SLAMR, which was identified in CA1 hippocampal neurons following contextual fear conditioning, exhibits activity-dependent localization along dendrites (124). Loss of SLAMR impairs dendritic complexity and spine plasticity, which ultimately affects fear memory consolidation. Importantly, SLAMR interacts with CaMKIIα, a kinase pivotal for synaptic plasticity, by using a conserved sequence that also governs its transport. Both ADEPTR and SLAMR respond to stimuli by moving into dendritic compartments—positions that facilitate localized interaction with key signaling molecules. However, the precise mechanisms by which these lncRNAs participate in signaling pathways are not clearly understood. For instance, while SLAMR appears to stabilize local CaMKIIα activity, does it act as a scaffold that arranges proteins for phosphorylation events, or does it recruit phosphatases to regulate kinase deactivation? Similarly, is ADEPTR directly modulating translation factors, or simply organizing cytoskeletal elements to optimize protein synthesis sites?

These findings underscore a broader challenge in ncRNA research: how do these transcripts govern translation and kinase activity at the molecular level? Knockdown or gain-of-function experiments often produce dramatic phenotypes—altered spine density, impaired axonal outgrowth, and changes in memory performance—implying that ncRNAs like SLAMR and Gas5 are integral to local protein synthesis (124, 125). However, it is unclear if these lncRNAs directly bind components of the ribosome, interacts with initiation factors, or modulates a kinase such as mTOR to orchestrate translation. These mechanistic gaps point to the need for subcellular CLIP-seq, cryo-ET of ribonucleoprotein particles, and cross-linking mass spectrometry to map how ncRNAs physically interface with the translational apparatus and kinase domains.

In search of regulators of RNA interactomes, recent studies indicate that cAMP/PKA signaling is a key mechanism by which synaptic activity reshapes ncRNA behavior. ADEPTR’s dendritic transport exemplifies how cAMP/PKA can direct ncRNA localization, but additional long-range interactions may also come into play. Transcription factors like CREB, which are activated by the cAMP cascade, could influence the transcriptional upregulation of specific lncRNAs, setting the stage for subsequent subcellular transport (124, 126). Conversely, PKA might phosphorylate RNA-binding proteins, altering their trafficking or binding affinities. Although direct biochemical evidence for these steps remains scarce, a critical question remains: can ncRNAs serve not only as downstream effectors of cAMP/PKA but also as upstream regulators that determine where and when protein phosphorylation cascades occur?

Building on this perspective of ncRNAs as both targets and drivers of signaling, LoNA provides a striking example of how activity-dependent shifts in nuclear-enriched lncRNAs can transform the cell’s translational landscape. Typically concentrated in the nucleolus, LoNA becomes downregulated under high neuronal firing, leading to an increase in ribosome biogenesis (127). Consequently, more ribosomes are available to be transported to synapses, boosting AMPA and NMDA receptor expression and enhancing synaptic responsiveness. Remarkably, LoNA deficiency not only augments long-term memory in wild-type mice but also rescues memory impairments in Alzheimer’s disease models (APP/PS1) (127). These findings suggest that activity-dependent changes in lncRNA abundance can operate as global regulatory switches for protein synthesis, linking local synaptic cues to broader translation programs critical for learning and memory.

An important factor, especially when considering rapid interactions required during activity in neurons, is the fact that the number of RNA molecules far exceeds the number of RBPs (128, 129). A single RNA molecule can interact with multiple RBPs simultaneously, sequentially, or in a mutually exclusive manner. In neurons, this balance becomes even more delicate. RBPs often localize to dendrites or axons where they engage select transcripts for local translation, while others shuttle between the nucleus and cytoplasm to control splicing or stability. These RBPs, similarly, can interact with various RNA molecules or preferentially interact with a particular subclass of these transcripts. Studies have shown that even the location at which RBPs bind can determine whether a transcript is stabilized, degraded, or translationally activated (12, 13). Binding profiles of various RBPs across nascent RNAs have revealed “RNA maps” which can now be used to predict whether an RBP will enhance or repress processes like alternative splicing or polyadenylation sites (129, 130, 131). Even with these maps, however, predictions of interactions remain difficult with most RNA-binding motifs spanning only 3 to 8 nucleotides (132, 133).

An emerging paradigm is that RNA structure can drive protein interaction specificity independently of sequence identity (134). Approximately 70% of characterized RBPs preferentially bind to short, linear sequence, whereas 30% recognize structured motifs such as stem-loops (135). Additionally, the number of protein contacts made by an RNA correlates with the connectivity of the encoded protein within interaction networks (134, 136). Simply put, more protein contacts with an mRNA means the encoded protein will interact more broadly or with more proteins once the transcript is translated. This feature becomes increasingly relevant in biomolecular condensates, where a RNA’s ability to bind 5 to 20 proteins per 100 nucleotides is immensely beneficial to its role as a molecular scaffold. While it seems that some RBPs might prefer more complex RNA structures, whether they exhibit different affinities for equally complex secondary structures remains a question of interest. In fact, certain highly structured RNAs have even been shown to disrupt pre-existing protein interactions. For example, HSP70 mRNA has been shown to compete with protein-protein interactions and leads to the release of specific proteins from aggregates (137).

Beyond traditional protein interactions, emerging evidence suggests that RNA may also interact with lipids, adding further complexity to the RNA interactome (138, 139, 140, 141, 142, 143). These interactions are often direct and have even been observed in ncRNAs, like tRNAs and lncRNAs like LINK-A, SNHG9, and LIPTER (144, 145, 146, 147, 148). These interactions could have profound implications for RNA transport, localization, and cellular signaling, yet their precise mechanisms remain poorly understood. RNA localization is often closely linked to vesicular transport, especially in neurons, yet whether RNA directly interacts with vesicle-associated lipids or whether these interactions influence transport decisions remains an open question.

Beyond transport, recent studies suggest that lipid membranes can modulate RNA catalytic activity. Experiments using the trans-acting R3C ligase ribozyme revealed that RNA-lipid interactions influence ribozyme activity in a concentration-dependent manner (140). These interactions, which occur primarily in guanine-rich sequences and double-stranded structures, demonstrate that lipids can act as platforms for riboregulation. Notably, there seems to be a relation between RNA secondary structure and the likelihood for these RNA-Lipid interactions. G-quadruplexes have been found to exhibit enhanced binding to both gel and fluid membranes, emphasizing potentially physiologically relevant roles (140).

Emerging techniques have begun to clarify how these interactions fluctuate in real-time. Techniques like CLIP-seq (149), RIP-seq (RNA immunoprecipitation sequencing), and related approaches such as iCLIP (150) or PAR-CLIP (151, 152, 153) yield high-resolution profiles of which RNAs bind to specific RBPs. As mentioned previously, coupling these immunoprecipitation methods with structural probing (*e*.*g*. IP-SHAPE-seq) could reveal how the secondary and tertiary structures of these RNAs shift upon RBP binding or under certain signaling conditions. For example, recent work demonstrates that cAMP stimulation changes RNase accessibility of the lncRNA Gas5 in RNP complexes in hippocampal neurons (154).

### Post-transcriptional regulation

The complexity of the RNA interactome is further shaped by post-transcriptional modifications that play major roles in transcript fate and subsequent interactions. These modifications can be large-scale, such as alternative splicing or alternative polyadenylation, or they can be site-specific chemical marks like N6-methyladenosine (m^6^A) or pseudouridine. Importantly, both mRNAs and ncRNAs are subject to a similar repertoire of processing and chemical changes, with profound consequences for RNA stability, localization, and intermolecular interactions.

Alternative splicing is one of the most prominent modifications at this level. While it plays a major role in protein diversity, it also modulates binding sites for RBPs and ncRNAs. The inclusion or exclusion of these key regulatory motifs has been shown to respond dynamically to cellular cues (155, 156). These splicing decisions are regulated by a network of small nuclear RNAs (snRNAs) that form the spliceosome complex (157, 158). However, other transcripts in the cell, including certain lncRNAs and miRNAs, may fine-tune these events by guiding splicing factors or influencing splice-site selection (159, 160, 161, 162). As a result, splicing emerges not as a simple gene-to-protein step but as a dynamic regulatory interface, where changes in isoform composition can alter a transcript’s entire fate and integration into the interactome.

Alternative polyadenylation (APA) serves as another key determinant of transcript interactions. APA generates isoforms with differing 3′ untranslated region (UTR) lengths, which in turn affect RBP and microRNA (miRNA) binding (163, 164). Shorter 3′UTRs often evade miRNA-mediated repression, leading to enhanced translation, whereas longer 3′UTRs create additional docking sites for regulatory factors. In neurons, where localized translation near synapses is crucial, 3′UTR length determines not just repression potential but subcellular fate. Longer 3′UTRs are often found in transcripts targeted to synaptic regions, perhaps to facilitate interaction with lncRNAs or retention in transport granules (165, 166). Thus, an RNA’s final 3′ configuration can profoundly affect its ability to engage with key regulators in the cytoplasm, subtly tuning gene expression programs in response to developmental or environmental cues (167). Importantly, APA intersects with alternative splicing, as splicing decisions can precede, or be modified by, polyadenylation site choice, combining to generate a vast repertoire of isoforms.

Beyond these large-scale processing events, nucleotide-level modifications play equally critical roles. m^6^A (N6-methyladenosine) marks are an abundant modification found across diverse transcripts (168). Recent studies suggest that this modification is not only highly specific but may also be under temporal control. m^6^A marks have been shown to accumulate most abundantly on steady-state RNAs that have completed splicing rather than newly synthesized RNAs (169). This observation indicates a progressive relationship between RNA processing and modification. Once installed by m^6^A methyltransferases, these marks help recruit specific “reader” proteins that direct transcripts toward particular fates—whether nuclear export, cytoplasmic translation, or decay. Interestingly, shorter 3′UTR isoforms have been reported to carry elevated levels of m^6^A, which further enhances their export and engagement with cytoplasmic RBPs (169). Neuronal m^6^A dynamics are tightly linked to plasticity and learning, with m^6^A readers such as YTHDF1 implicated in memory consolidation and local translation in dendrites (170, 171). As a result, changing poly(A) sites or splicing patterns can modify how extensively a transcript is m^6^A-marked, illustrating how interplay between different post-transcriptional layers shapes global RNA distribution and availability for interactions.

Several other chemical modifications contribute to this evolving “epitranscriptome”. Pseudouridylation and 2′-O-methylation can alter base-pairing stability or RBP recognition (172), affecting both mRNAs and ncRNAs. By subtly tweaking tertiary folds or surface chemistries, these marks can strengthen or weaken certain RNA-protein interactions, further refining the way transcripts integrate into the cell’s regulatory circuits. While some modifications may be installed co-transcriptionally, others occur later in the RNA lifespan, enabling fine-tuned responses to external stimuli or metabolic states. For example, certain ncRNAs appear to gain 2′-O-methylation selectively under stress, modulating their capacity to scaffold nuclear condensates or sequester proteins in stress granules (173).

Intriguingly, transfer RNAs also undergo extensive modification, and modified tRNAs appear to be unusually abundant in the brain (174, 175, 176). This enrichment suggests a specialized role for tRNA modifications in neuronal physiology, possibly to meet the high demands for local protein synthesis at synapses. Modified tRNAs may help buffer against codon misreading, modulate the pace of elongation, or fine-tune translation in response to neural activity. Given that neurons rely on rapid, spatially restricted bursts of protein synthesis to support processes like plasticity and memory, the heightened prevalence of modified tRNAs in the brain hints at a critical and perhaps underexplored layer of post-transcriptional regulation in the nervous system.

Another particularly novel modification is the covalent attachment of glycans to RNA to form glycoRNAs. These modified transcripts have been found to localize to the cell surface to interact with glycan-binding proteins, such as Siglecs, that play major roles in immune-neural signaling (177). Nonetheless, the specifics of glycoRNA biogenesis and function remain poorly understood. It is plausible that, just as in protein-protein interactions, glycosylation may act as a modifier of RNA-protein networks. glycoRNAs could provide docking sites for glycan-binding proteins in a sequence-independent manner, which can alter the specificity of RBP interactions. Alternatively, glycans may serve as a quality control and localization mechanism by marking specific ncRNAs for trafficking to the cell surface, retention in membrane-associated compartments, or degradation. In neurons, glycoRNA modification could serve as a marker for synaptic localization, vesicular trafficking, or degradation. This idea raises an intriguing question: Does glycosylation of RNAs depend on sequence motifs, structural features, or co-transcriptional associations with glycosylation enzymes?

It is crucial to recognize that these post-transcriptional modifications interconnect with one another. Transcripts that undergo particular splicing events may accumulate distinct sets of nucleotide modifications; RNAs that are hypermethylated at m^6^A sites could be preferentially packaged into exosomes for export; and glycosylation might intersect with cytoplasmic capping or uridylation to control an RNA’s final destination. By reframing transcripts as fluid rather than static sequences, we begin to appreciate how the post-transcriptional challenge expands the functional repertoire of RNA. Indeed, each modification or processing decision can shift a transcript’s conformation, subcellular location, or half-life, thereby altering its potential to recruit or repel other RNAs and proteins. Synaptic transcripts may require unique splicing isoforms to engage local readers, axonal RNAs might be pseudouridylated to prevent premature degradation, and lncRNAs modulating plasticity may acquire or lose modifications based on circadian phase or behavioral state.

To advance past the post-transcriptional challenge, improved sequencing and chemical tools for detecting RNA modifications are paramount. Techniques such as methylated RNA immunoprecipitation sequencing (MeRIP-seq) for mapping m^6^A or Ribo-Meth-seq for 2′-O-methylation provide global maps but often lack single-nucleotide resolution that can allow for proper quantification. At a basic level, combining site-specific mutagenesis (*e*.*g*. *via* CRISPR base editors) with phenotypic assays could reveal whether a modification is dispensable or essential. The next frontier likely involves high-throughput functional screens, in which libraries of mutated transcripts with or without specific modifications are assayed for changes in RBP and ncRNA binding or localization patterns. For glycoRNAs, advanced click chemistry reagents could enable pull-down of glycoRNAs, clarifying which sequences are preferentially modified and how that influences RBP accessibility. By bridging these efforts with the projects described in the previous four challenges, we can expect to uncover how post-transcriptional modifications help orchestrate the wide-ranging and continually evolving repertoire of RNA function.

## ncRNA-mediated regulation in neuropsychiatric disorders

**Figure 3:**
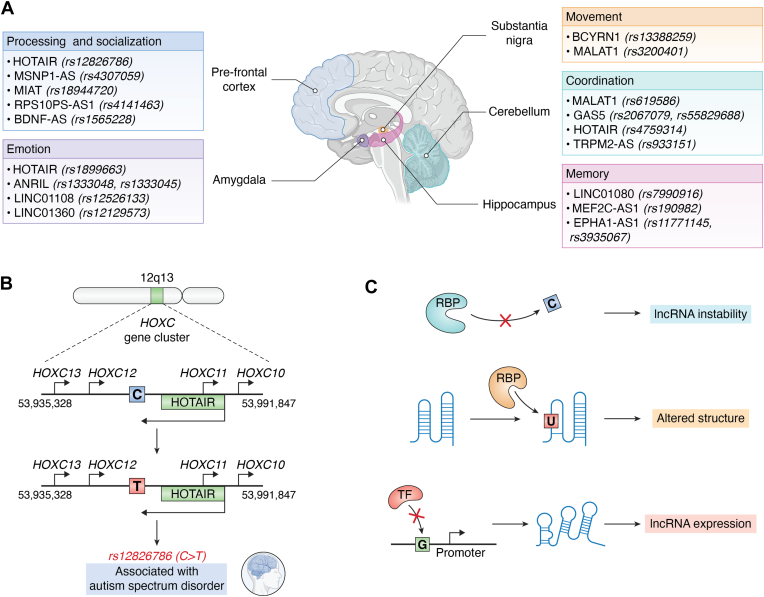
**lncRNA SNPs Implicated in neuropsychiatric disorders**. *A*, single-nucleotide polymorphisms (SNPs) within lncRNAs contribute to neuropsychiatric disorders by disrupting processes like social behavior, emotion, movement, coordination, and memory. For example, SNPs in HOTAIR, MSNP1-AS, MIAT, RPS10PS-AS1, and BDNF-AS are linked to autism and schizophrenia (178, 179, 180, 181, 182). SNPs in HOTAIR, ANRIL, LINC01108, and LINC01360 have been associated with major depressive disorder (152, 183, 184, 185) SNPs in BCYRN1 and MALAT1 are implicated in Parkinson’s Disease (186, 187). SNPs in MALAT1, GAS5, HOTAIR, and TRPM2-AS are linked to multiple sclerosis (188, 189, 190, 191, 192, 193). Lastly, SNPs in LINC01080, MEF2C-AS1, and EPHA1-AS1 are implicated in Alzheimer’s Disease (194, 195, 196, 197). *B*, schematic of example C > T SNP rs12826786 in lncRNA HOTAIR, which has been implicated in ASD. *C*, Three example mechanisms by which SNPs in lncRNAs can have functional implications: (1) lncRNA instability - SNPs can prevent the binding of RBPs that promote stability of these transcripts, (2) altered structure – lncRNA structure can change due to position of SNP which can interfere with downstream interactions, (3) lncRNA expression – SNPs in the promotor region of genes coding for lncRNAs can influence lncRNA levels and interfere with function.

One of the most prominent lines of evidence for the critical roles of ncRNAs in neuronal function is the clear association of ncRNA mutations with various neuropsychiatric disorders (Fig. 3). Although these transcripts do not directly encode proteins, their ability to shape gene expression and intracellular signaling can be profoundly altered by even minor nucleotide changes—leading to significant behavioral and cognitive deficits. Such findings underscore how intimately ncRNAs are involved in establishing and maintaining the neural circuits that underlie complex brain functions.

While several reviews have aimed to dissect the molecular mechanisms of neuropsychiatric disorders, this discussion focuses on highlighting specific mutations in lncRNAs associated with these conditions (198, 199, 200). In Figure 3, we highlight a selection of common single nucleotide polymorphisms (SNPs) in lncRNAs that have been implicated in five broad neurological processes: (1) processing and socialization - commonly linked to autism and schizophrenia, (2) emotion - major depressive disorder, (3) movement - Parkinson’s disease, (4) coordination - multiple sclerosis, and (5) memory - Alzheimer’s disease. These genetic variations can alter the structure and function of lncRNAs, potentially disrupting their regulatory roles in the brain. For example, HOTAIR harbors the SNP rs12826786, associated with autism and schizophrenia (178), and Gas5 contains rs2067079, implicated in multiple sclerosis (188, 189). Interestingly, early-life stress has been shown to enhance the expression of Gas5, whose elevated levels are also linked to anxiety and spatial memory deficits (201). Mutations in these lncRNAs can alter secondary structures or disrupt binding motifs, reshaping how they modulate chromatin structure, transcription factor recruitment, or RNA-protein complex formation. For example, SNP rs217727 has been shown to alter the secondary structure of lncRNA H19 and is associated with dilated cardiomyopathy (202). Similarly, these perturbations can manifest in regions of the brain critical to the implicated functions—such as the prefrontal cortex, hippocampus, amygdala, cerebellum, or substantia nigra.

Other examples include the SNP rs4307059 in MSNP1-AS, which is encoded by the opposite (antisense) strand of MSNP1 and has been linked to autism spectrum disorder (179). LINC01108 and LINC01360 polymorphisms are reported in depressive disorders, wherein their altered expression correlates with dysregulated gene networks supporting mood and stress responses (183, 184). At a mechanistic level, these mutations may misdirect splicing regulators or scaffold proteins, prompting broad transcriptomic imbalances.

Beyond lncRNAs, mutations in miRNAs have also been linked to neuropsychiatric disorders. miR-34b/c is upregulated in patients with schizophrenia, and its increased expression correlates with deficits in synaptic plasticity and cognitive function (203). miRNAs regulate gene expression by binding to complementary sequences in target mRNAs, and mutations that impair miRNA function can result in the dysregulation of genes critical for synaptic function and neuronal connectivity. miR-137, another miRNA implicated in schizophrenia, regulates key neuronal genes such as CACNA1C, which encodes a calcium channel subunit (204, 205). SNPs in the miR-137 gene can alter its processing or target gene specificity, potentially contributing to the development of psychiatric disorders by disrupting synaptic plasticity (206, 207, 208).

As our understanding of the molecular mechanisms underlying these mutations grows, it has become clear that ncRNAs are not only essential for normal brain function but also pivotal in the development of neuropsychiatric conditions. The circuit-level impact of a single ncRNA mutation illustrates how localized molecular perturbations can reverberate through broader neuronal networks. Exploring the relationship between ncRNA mutations and mental health could provide valuable insights into novel therapeutic approaches for these complex disorders.

A particularly promising therapeutic focus involves natural antisense transcripts (NATs), an abundant subclass of lncRNAs transcribed from the strand opposite protein-coding or non-coding genes. Since NATs can regulate sense-strand transcription, splicing, stability, and translation, a single-nucleotide change that alters the extent or structure of sense–antisense overlap can propagate dysfunction across multiple regulatory layers (209). This expectation is supported by studies on the Bdnf-AS knockout mouse, in which deletion of 6 kb upstream of Bdnf-AS boosts endogenous BDNF protein and enhances exercise-induced memory (210). Furthermore, examples of SNPs in NATs like MSNP1-AS and MAPT-AS1 are shown in Figure 3 in the context of neuropsychiatric disorders. This work highlights both the pathogenic potential of NAT dysregulation and the feasibility of targeting antisense loci to restore neuronal resilience.

Translating these genetic insights into therapy is increasingly practical due to advances in nucleic-acid-based therapeutics (NBTs) like antisense oligonucleotides, siRNAs, splice-switching ASOs, and CRISPR guides. Chemical stabilization, ligand conjugation, and nanoparticle or viral delivery systems are now being optimized to traverse the blood–brain barrier, allowing systemic administration rather than direct intracranial injection (211). Such platforms could, in principle, silence toxic RNAs, correct a riboSNitch in a lncRNA, or replace a dysfunctional miRNA, offering precise interventions for disorders that have long eluded conventional protein-centric drugs.

## Final remarks

Throughout this review, we have highlighted the expanding roles of RNA far beyond its classical function as an intermediary between DNA and protein. The field has moved past the early frameworks of molecular biology to recognize RNA as a central player in scaffolding, catalysis, biomolecular condensate formation, and post-transcriptional regulation. Together, these discoveries point to an interactionist perspective of RNA biology—where transcripts function as dynamic nodes within intricate molecular networks.

In revisiting the central questions posed at the outset, the evidence discussed makes clear that RNA interactions are directed by structural motifs, chemical modifications, and spatial arrangements within the cell. These principles acquire additional layers of complexity in neurons, where activity-dependent and spatiotemporal regulation of ncRNAs is indispensable for synaptic plasticity. While our focus here has been on neuronal systems, comparable specificity is likely required in other specialized contexts, such as immune or stem cells. A pressing challenge remains to determine how the RNA interactome adapts to the demands of distinct cell types, and whether generalizable principles can be identified across cellular systems.

Despite considerable progress, key questions remain. We have outlined five major challenges facing the field and emphasized how emerging technologies—ranging from single-molecule high-resolution imaging to advanced computational modeling—offer new opportunities to decode these complex networks. Yet, mechanistic insights into how RNA interactions are prioritized and resolved in real time within living cells remain limited. Addressing these gaps will require integrating single-cell and systems-level approaches to refine conceptual frameworks and accelerate translational applications. Ultimately, the study of RNA biology continues to redefine the molecular logic of the cell.

## Conflict of interest

The authors declare that they have no conflicts of interest with the contents of this article.

## References

[1] Crick F.H. (1958). On protein synthesis. Symp. Soc. Exp. Biol..

[2] Crick F. (1970). Central dogma of molecular biology. Nature.

[3] Eddy S.R. (2001). Non-coding RNA genes and the modern RNA world. Nat. Rev. Genet..

[4] Mattick J.S., Makunin I.V. (2006). Non-coding RNA. Hum. Mol. Genet..

[5] Esteller M. (2011). Non-coding RNAs in human disease. Nat. Rev. Genet..

[6] Shahzad U., Krumholtz S., Rutka J.T., Das S. (2021). Noncoding RNAs in glioblastoma: emerging biological concepts and potential therapeutic implications. Cancers (Basel).

[7] Kaikkonen M.U., Lam M.T., Glass C.K. (2011). Non-coding RNAs as regulators of gene expression and epigenetics. Cardiovasc. Res..

[8] Mattick J.S., Amaral P.P., Carninci P., Carpenter S., Chang H.Y., Chen L.L. (2023). Long non-coding RNAs: definitions, functions, challenges and recommendations. Nat. Rev. Mol. Cell. Biol..

[9] Hamel K., Moncada E.L., Sheeler C., Rosa J.G., Gilliat S., Zhang Y. (2024). Cerebellar heterogeneity and selective vulnerability in spinocerebellar ataxia type 1 (SCA1). Neurobiol. Dis..

[10] Nussbacher J.K., Tabet R., Yeo G.W., Lagier-Tourenne C. (2019). Disruption of RNA metabolism in neurological diseases and emerging therapeutic interventions. Neuron.

[11] Kornienko I.V., Aramova O.Y., Tishchenko A.A., Rudoy D.V., Chikindas M.L. (2024). RNA stability: a review of the role of structural features and environmental conditions. Molecules.

[12] Corley M., Burns M.C., Yeo G.W. (2020). How RNA-binding proteins interact with RNA: molecules and mechanisms. Mol. Cell..

[13] Li W., Deng X., Chen J. (2022). RNA-binding proteins in regulating mRNA stability and translation: roles and mechanisms in cancer. Semin. Cancer. Biol..

[14] Mustoe A.M., Busan S., Rice G.M., Hajdin C.E., Peterson B.K., Ruda V.M. (2018). Pervasive regulatory functions of mRNA structure revealed by high-resolution SHAPE probing. Cell.

[15] Bocobza S., Adato A., Mandel T., Shapira M., Nudler E., Aharoni A. (2007). Riboswitch-dependent gene regulation and its evolution in the plant kingdom. Genes Dev..

[16] Widagdo J., Anggono V. (2018). The m^6^A-epitranscriptomic signature in neurobiology: from neurodevelopment to brain plasticity. J. Neurochem..

[17] Ling S.C., Polymenidou M., Cleveland D.W. (2013). Converging mechanisms in ALS and FTD: disrupted RNA and protein homeostasis. Neuron.

[18] Carrasco M., Rabaneda L.G., Murillo-Carretero M., Ortega-Martínez S., Martínez-Chantar M.L., Woodhoo A. (2014). Glycine N-methyltransferase expression in the hippocampus and its role in neurogenesis and cognitive performance. Hippocampus.

[19] Sánchez-Ramírez E., Ung T.P.L., Stringari C., Aguilar-Arnal L. (2024). Emerging functional connections between metabolism and epigenetic remodeling in neural differentiation. Mol. Neurobiol..

[20] Jadavji N.M., Deng L., Malysheva O., Caudill M.A., Rozen R. (2015). MTHFR deficiency or reduced intake of folate or choline in pregnant mice results in impaired short-term memory and increased apoptosis in the hippocampus of wild-type offspring. Neuroscience.

[21] Yaliwal L.V., Desai R.M. (2012). Methylenetetrahydrofolate reductase mutations, a genetic cause for familial recurrent neural tube defects. Indian J. Hum. Genet..

[22] Mendelsohn R., Garcia G.C., Bartol T.M., Lee C.T., Khandelwal P., Liu E. (2022). Morphological principles of neuronal mitochondria. J. Comp. Neurol..

[23] López-Domenech G., Kittler J.T. (2023). Mitochondrial regulation of local supply of energy in neurons. Curr. Opin. Neurobiol..

[24] Sheng Z.H., Cai Q. (2012). Mitochondrial transport in neurons: impact on synaptic homeostasis and neurodegeneration. Nat. Rev. Neurosci..

[25] Pushpalatha K.V., Besse F. (2019). Local translation in axons: when membraneless RNP granules meet membrane-mound organelles. Front Mol. Biosci..

[26] Cioni J.M., Lin J.Q., Holtermann A.V., Koppers M., Jakobs M.A.H., Azizi A. (2019). Late endosomes act as mRNA translation platforms and sustain mitochondria in axons. Cell.

[27] Fenton A.R., Peng R., Bond C., Hugelier S., Lakadamyali M., Chang Y.W. (2024). FMRP regulates MFF translation to locally direct mitochondrial fission in neurons. Nat. Cell. Biol..

[28] Wheeler H.B., Madrigal A.A., Chaim I.A. (2024). Mapping the future of oxidative RNA damage in neurodegeneration: rethinking the status quo with new tools. Proc. Natl. Acad. Sci. U. S. A..

[29] Howe M., Patani R. (2023). Nonsense-mediated mRNA decay in neuronal physiology and neurodegeneration. Trends Neurosci..

[30] Son H.G., Seo M., Ham S., Hwang W., Lee D., An S.W. (2017). RNA surveillance via nonsense-mediated mRNA decay is crucial for longevity in daf-2/insulin/IGF-1 mutant C. elegans. Nat. Commun..

[31] Zuniga G., Levy S., Ramirez P., De Mange J., Gonzalez E., Gamez M. (2023). Tau-induced deficits in nonsense-mediated mRNA decay contribute to neurodegeneration. Alzheimers Dement.

[32] Tarpey P.S., Raymond F.L., Nguyen L.S., Rodriguez J., Hackett A., Vandeleur L. (2007). Mutations in UPF3B, a member of the nonsense-mediated mRNA decay complex, cause syndromic and nonsyndromic mental retardation. Nat. Genet..

[33] Boczonadi V., Müller J.S., Pyle A., Munkley J., Dor T., Quartararo J. (2014). EXOSC8 mutations alter mRNA metabolism and cause hypomyelination with spinal muscular atrophy and cerebellar hypoplasia. Nat. Commun..

[34] Cao X., Yeo G., Muotri A.R., Kuwabara T., Gage F.H. (2006). Noncoding RNAs in the mammalian central nervous system. Annu. Rev. Neurosci..

[35] Salvatori B., Biscarini S., Morlando M. (2020). Non-coding RNAs in nervous system development and disease. Front. Cell Dev. Biol..

[36] Briggs J.A., Wolvetang E.J., Mattick J.S., Rinn J.L., Barry G. (2015). Mechanisms of long non-coding RNAs in mammalian nervous system development, plasticity, disease, and evolution. Neuron.

[37] Bernard D., Prasanth K.V., Tripathi V., Colasse S., Nakamura T., Xuan Z. (2010). A long nuclear-retained non-coding RNA regulates synaptogenesis by modulating gene expression. EMBO J..

[38] Raveendra B.L., Swarnkar S., Avchalumov Y., Liu X.A., Grinman E., Badal K. (2018). Long noncoding RNA GM12371 acts as a transcriptional regulator of synapse function. Proc. Natl. Acad. Sci. U. S. A..

[39] Liau W.S., Zhao Q., Bademosi A., Gormal R.S., Gong H., Marshall P.R. (2023). Fear extinction is regulated by the activity of long noncoding RNAs at the synapse. Nat. Commun..

[40] An H., Williams N.G., Shelkovnikova T.A. (2018). NEAT1 and paraspeckles in neurodegenerative diseases: a missing lnc found?. Noncoding RNA Res..

[41] Kukharsky M.S., Ninkina N.N., An H., Telezhkin V., Wei W., Meritens C.R. (2020). Long non-coding RNA Neat1 regulates adaptive behavioural response to stress in mice. Transl Psychiatry.

[42] Hansen K.F., Sakamoto K., Wayman G.A., Impey S., Obrietan K. (2010). Transgenic miR132 alters neuronal spine density and impairs novel object recognition memory. PLoS One.

[43] Konopka W., Kiryk A., Novak M., Herwerth M., Parkitna J.R., Wawrzyniak M. (2010). MicroRNA loss enhances learning and memory in mice. J. Neurosci..

[44] Fallon L., Jones A.N. (2024). Alternative conformations of lncRNAs identified through structural deconvolution of SHAPE- and DMS-MaP datasets. bioRxiv.

[45] Chang Y.Z., Chai R.C., Pang B., Chang X., An S.Y., Zhang K.N. (2021). METTL3 enhances the stability of MALAT1 with the assistance of HuR via m^6^A modification and activates NF-κB to promote the malignant progression of IDH-wildtype glioma. Cancer Lett..

[46] Panneerdoss S., Eedunuri V.K., Yadav P., Timilsina S., Rajamanickam S., Viswanadhapalli S. (2018). Cross-talk among writers, readers, and erasers of m. Sci. Adv..

[47] Derrien T., Johnson R., Bussotti G., Tanzer A., Djebali S., Tilgner H. (2012). The GENCODE v7 catalog of human long noncoding RNAs: analysis of their gene structure, evolution, and expression. Genome Res..

[48] Kaushik K., Leonard V.E., Kv S., Lalwani M.K., Jalali S., Patowary A. (2013). Dynamic expression of long non-coding RNAs (lncRNAs) in adult zebrafish. PLoS One.

[49] Mercer T.R., Dinger M.E., Sunkin S.M., Mehler M.F., Mattick J.S. (2008). Specific expression of long noncoding RNAs in the mouse brain. Proc. Natl. Acad. Sci. U. S. A..

[50] Kadakkuzha B.M., Liu X.A., McCrate J., Shankar G., Rizzo V., Afinogenova A. (2015). Transcriptome analyses of adult mouse brain reveal enrichment of lncRNAs in specific brain regions and neuronal populations. Front. Cell Neurosci..

[51] Schratt G.M., Tuebing F., Nigh E.A., Kane C.G., Sabatini M.E., Kiebler M. (2006). A brain-specific microRNA regulates dendritic spine development. Nature.

[52] Peng C., Li L., Zhang M.D., Bengtsson Gonzales C., Parisien M., Belfer I. (2017). miR-183 cluster scales mechanical pain sensitivity by regulating basal and neuropathic pain genes. Science.

[53] Liau W.S., Samaddar S., Banerjee S., Bredy T.W. (2021). On the functional relevance of spatiotemporally-specific patterns of experience-dependent long noncoding RNA expression in the brain. RNA Biol..

[54] Butler A.A., Johnston D.R., Kaur S., Lubin F.D. (2019). Long noncoding RNA NEAT1 mediates neuronal histone methylation and age-related memory impairment. Sci. Signal..

[55] Barry G., Briggs J.A., Hwang D.W., Nayler S.P., Fortuna P.R., Jonkhout N. (2017). The long non-coding RNA NEAT1 is responsive to neuronal activity and is associated with hyperexcitability states. Sci. Rep..

[56] Jiang T., Cai Z., Ji Z., Zou J., Liang Z., Zhang G. (2020). The lncRNA MALAT1/miR-30/Spastin axis regulates hippocampal neurite outgrowth. Front. Cell Neurosci..

[57] Lee Y.S., Nakahara K., Pham J.W., Kim K., He Z., Sontheimer E.J. (2004). Distinct roles for Drosophila Dicer-1 and Dicer-2 in the siRNA/miRNA silencing pathways. Cell.

[58] Hansen T.B., Jensen T.I., Clausen B.H., Bramsen J.B., Finsen B., Damgaard C.K. (2013). Natural RNA circles function as efficient microRNA sponges. Nature.

[59] Rahimi K., Færch Nielsen A., Venø M.T., Kjems J. (2021). Nanopore long-read sequencing of circRNAs. Methods.

[60] Taylor S.C., Laperriere G., Germain H. (2017). Droplet digital PCR versus qPCR for gene expression analysis with low abundant targets: from variable nonsense to publication quality data. Sci. Rep..

[61] Taruttis F., Feist M., Schwarzfischer P., Gronwald W., Kube D., Spang R. (2017). External calibration with drosophila whole-cell spike-ins delivers absolute mRNA fold changes from human RNA-Seq and qPCR data. Biotechniques.

[62] Callens C., Bidard F.C., Curto-Taribo A., Trabelsi-Grati O., Melaabi S., Delaloge S. (2022). Real-time detection of ESR1 mutation in blood by droplet digital PCR in the PADA-1 tial: feasibility and cross-validation with NGS. Anal. Chem..

[63] Fang S., Zhang L., Guo J., Niu Y., Wu Y., Li H. (2018). NONCODEV5: a comprehensive annotation database for long non-coding RNAs. Nucleic Acids Res..

[64] Crossley M.P., Bocek M., Cimprich K.A. (2019). R-Loops as cellular regulators and genomic threats. Mol. Cell.

[65] Niehrs C., Luke B. (2020). Regulatory R-loops as facilitators of gene expression and genome stability. Nat. Rev. Mol. Cell Biol..

[66] Tholstrup J., Oddershede L.B., Sørensen M.A. (2012). mRNA pseudoknot structures can act as ribosomal roadblocks. Nucleic Acids Res..

[67] Kharel P., Ivanov P. (2024). RNA G-quadruplexes and stress: emerging mechanisms and functions. Trends Cell Biol..

[68] Thomas M., White R.L., Davis R.W. (1976). Hybridization of RNA to double-stranded DNA: formation of R-loops. Proc. Natl. Acad. Sci. U. S. A..

[69] Drolet M., Phoenix P., Menzel R., Massé E., Liu L.F., Crouch R.J. (1995). Overexpression of RNase H partially complements the growth defect of an Escherichia coli delta topA mutant: R-loop formation is a major problem in the absence of DNA topoisomerase I. Proc. Natl. Acad. Sci. U. S. A..

[70] Serra-Cardona A., Zhang Z. (2018). Replication-coupled nucleosome assembly in the passage of epigenetic information and cell identity. Trends Biochem. Sci..

[71] Chang H.W., Pandey M., Kulaeva O.I., Patel S.S., Studitsky V.M. (2016). Overcoming a nucleosomal barrier to replication. Sci. Adv..

[72] Gan W., Guan Z., Liu J., Gui T., Shen K., Manley J.L. (2011). R-loop-mediated genomic instability is caused by impairment of replication fork progression. Genes. Dev..

[73] Aguilera A., García-Muse T. (2012). R loops: from transcription byproducts to threats to genome stability. Mol. Cell.

[74] Gong D., Wang L., Zhou H., Gao J., Zhang W., Zheng P. (2023). Long noncoding RNA Lnc530 localizes on R-loops and regulates R-loop formation and genomic stability in mouse embryonic stem cells. Stem. Cell Rep..

[75] Cuartas J., Gangwani L. (2022). R-loop mediated DNA damage and impaired DNA repair in spinal muscular atrophy. Front. Cell Neurosci..

[76] Gellert M., Lipsett M.N., Davies D.R. (1962). Helix formation by guanylic acid. Proc. Natl. Acad. Sci. U. S. A..

[77] Georgakopoulos-Soares I., Parada G.E., Wong H.Y., Medhi R., Furlan G., Munita R. (2022). Alternative splicing modulation by G-quadruplexes. Nat. Commun..

[78] Rhodes D., Lipps H.J. (2015). G-quadruplexes and their regulatory roles in biology. Nucleic Acids Res..

[79] Smith J.S., Chen Q., Yatsunyk L.A., Nicoludis J.M., Garcia M.S., Kranaster R. (2011). Rudimentary G-quadruplex-based telomere capping in Saccharomyces cerevisiae. Nat. Struct. Mol. Biol..

[80] Zhang Y., Yang M., Duncan S., Yang X., Abdelhamid M.A.S., Huang L. (2019). G-quadruplex structures trigger RNA phase separation. Nucleic Acids Res..

[81] Asamitsu S., Yabuki Y., Matsuo K., Kawasaki M., Hirose Y., Kashiwazaki G. (2023). RNA G-quadruplex organizes stress granule assembly through DNAPTP6 in neurons. Sci. Adv..

[82] Matsuo K., Asamitsu S., Maeda K., Suzuki H., Kawakubo K., Komiya G. (2024). RNA G-quadruplexes form scaffolds that promote neuropathological α-synuclein aggregation. Cell.

[83] Pleij C.W., Rietveld K., Bosch L. (1985). A new principle of RNA folding based on pseudoknotting. Nucleic Acids Res..

[84] Rietveld K., Pleij C.W., Bosch L. (1983). Three-dimensional models of the tRNA-like 3' termini of some plant viral RNAs. EMBO J..

[85] Kolk M.H., van der Graaf M., Wijmenga S.S., Pleij C.W., Heus H.A., Hilbers C.W. (1998). NMR structure of a classical pseudoknot: interplay of single- and double-stranded RNA. Science.

[86] Su L., Chen L., Egli M., Berger J.M., Rich A. (1999). Minor groove RNA triplex in the crystal structure of a ribosomal frameshifting viral pseudoknot. Nat. Struct. Biol..

[87] Namy O., Moran S.J., Stuart D.I., Gilbert R.J., Brierley I. (2006). A mechanical explanation of RNA pseudoknot function in programmed ribosomal frameshifting. Nature.

[88] Farabaugh P.J. (1996). Programmed translational frameshifting. Microbiol. Rev..

[89] Mironov A.S., Gusarov I., Rafikov R., Lopez L.E., Shatalin K., Kreneva R.A. (2002). Sensing small molecules by nascent RNA: a mechanism to control transcription in bacteria. Cell.

[90] Winkler W., Nahvi A., Breaker R.R. (2002). Thiamine derivatives bind messenger RNAs directly to regulate bacterial gene expression. Nature.

[91] Nahvi A., Sudarsan N., Ebert M.S., Zou X., Brown K.L., Breaker R.R. (2002). Genetic control by a metabolite binding mRNA. Chem. Biol..

[92] Quinodoz S., Guttman M. (2014). Long noncoding RNAs: an emerging link between gene regulation and nuclear organization. Trends Cell Biol..

[93] Dixon-McDougall T., Brown C.J. (2022). Multiple distinct domains of human XIST are required to coordinate gene silencing and subsequent heterochromatin formation. Epigenetics Chromatin.

[94] Chu C., Zhang Q.C., da Rocha S.T., Flynn R.A., Bharadwaj M., Calabrese J.M. (2015). Systematic discovery of Xist RNA binding proteins. Cell.

[95] Hirose T., Yamazaki T., Nakagawa S. (2019). Molecular anatomy of the architectural NEAT1 noncoding RNA: the domains, interactors, and biogenesis pathway required to build phase-separated nuclear paraspeckles. Wiley Interdiscip. Rev. RNA.

[96] Clemson C.M., Hutchinson J.N., Sara S.A., Ensminger A.W., Fox A.H., Chess A. (2009). An architectural role for a nuclear noncoding RNA: NEAT1 RNA is essential for the structure of paraspeckles. Mol. Cell.

[97] Nakagawa S., Ip J.Y., Shioi G., Tripathi V., Zong X., Hirose T. (2012). MALAT1 is not an essential component of nuclear speckles in mice. RNA.

[98] Roden C., Gladfelter A.S. (2021). RNA contributions to the form and function of biomolecular condensates. Nat. Rev. Mol. Cell Biol..

[99] Rybak-Wolf A., Stottmeister C., Glažar P., Jens M., Pino N., Giusti S. (2015). Circular RNAs in the mammalian brain are highly abundant, conserved, and dynamically expressed. Mol. Cell.

[100] Memczak S., Jens M., Elefsinioti A., Torti F., Krueger J., Rybak A. (2013). Circular RNAs are a large class of animal RNAs with regulatory potency. Nature.

[101] You X., Vlatkovic I., Babic A., Will T., Epstein I., Tushev G. (2015). Neural circular RNAs are derived from synaptic genes and regulated by development and plasticity. Nat. Neurosci..

[102] Giusti S.A., Pino N.S., Pannunzio C., Ogando M.B., Armando N.G., Garrett L. (2024). A brain-enriched circular RNA controls excitatory neurotransmission and restricts sensitivity to aversive stimuli. Sci. Adv..

[103] Banani S.F., Lee H.O., Hyman A.A., Rosen M.K. (2017). Biomolecular condensates: organizers of cellular biochemistry. Nat. Rev. Mol. Cell Biol..

[104] Bond C.S., Fox A.H. (2009). Paraspeckles: nuclear bodies built on long noncoding RNA. J. Cell Biol..

[105] Quinodoz S.A., Jachowicz J.W., Bhat P., Ollikainen N., Banerjee A.K., Goronzy I.N. (2021). RNA promotes the formation of spatial compartments in the nucleus. Cell.

[106] Miao H., Wu F., Li Y., Qin C., Zhao Y., Xie M. (2022). MALAT1 modulates alternative splicing by cooperating with the splicing factors PTBP1 and PSF. Sci. Adv..

[107] Talkish J., May G., Lin Y., Woolford J.L., McManus C.J. (2014). Mod-seq: high-throughput sequencing for chemical probing of RNA structure. RNA.

[108] Watters K.E., Strobel E.J., Yu A.M., Lis J.T., Lucks J.B. (2016). Cotranscriptional folding of a riboswitch at nucleotide resolution. Nat. Struct. Mol. Biol..

[109] Sun L., Fazal F.M., Li P., Broughton J.P., Lee B., Tang L. (2019). RNA structure maps across mammalian cellular compartments. Nat. Struct. Mol. Biol..

[110] Zubradt M., Gupta P., Persad S., Lambowitz A.M., Weissman J.S., Rouskin S. (2017). DMS-MaPseq for genome-wide or targeted RNA structure probing in vivo. Nat. Methods.

[111] Bohn P., Gribling-Burrer A.S., Ambi U.B., Smyth R.P. (2023). Nano-DMS-MaP allows isoform-specific RNA structure determination. Nat. Methods.

[112] Liu X., Huang H., Karbstein K. (2022). Using DMS-MaPseq to uncover the roles of DEAD-box proteins in ribosome assembly. Methods.

[113] Spitale R.C., Crisalli P., Flynn R.A., Torre E.A., Kool E.T., Chang H.Y. (2013). RNA SHAPE analysis in living cells. Nat. Chem. Biol..

[114] Spitale R.C., Flynn R.A., Zhang Q.C., Crisalli P., Lee B., Jung J.W. (2015). Structural imprints in vivo decode RNA regulatory mechanisms. Nature.

[115] Guttman M., Amit I., Garber M., French C., Lin M.F., Feldser D. (2009). Chromatin signature reveals over a thousand highly conserved large non-coding RNAs in mammals. Nature.

[116] Pang K.C., Frith M.C., Mattick J.S. (2006). Rapid evolution of noncoding RNAs: lack of conservation does not mean lack of function. Trends Genet..

[117] Uroda T., Anastasakou E., Rossi A., Teulon J.M., Pellequer J.L., Annibale P. (2019). Conserved Pseudoknots in lncRNA MEG3 are essential for stimulation of the p53 pathway. Mol. Cell.

[118] Cheah M.T., Wachter A., Sudarsan N., Breaker R.R. (2007). Control of alternative RNA splicing and gene expression by eukaryotic riboswitches. Nature.

[119] Azzalin C.M., Reichenbach P., Khoriauli L., Giulotto E., Lingner J. (2007). Telomeric repeat containing RNA and RNA surveillance factors at mammalian chromosome ends. Science.

[120] Bautista-Sánchez D., Arriaga-Canon C., Pedroza-Torres A., De La Rosa-Velázquez I.A., González-Barrios R., Contreras-Espinosa L. (2020). The promising role of miR-21 as a cancer biomarker and its importance in RNA-based therapeutics. Mol. Ther. Nucleic Acids.

[121] Nawrocki E.P., Kolbe D.L., Eddy S.R. (2009). Infernal 1.0: inference of RNA alignments. Bioinformatics.

[122] Griffiths-Jones S., Bateman A., Marshall M., Khanna A., Eddy S.R. (2003). Rfam: an RNA family database. Nucleic Acids Res..

[123] Grinman E., Nakahata Y., Avchalumov Y., Espadas I., Swarnkar S., Yasuda R. (2021). Activity-regulated synaptic targeting of lncRNA ADEPTR mediates structural plasticity by localizing Sptn1 and AnkB in dendrites. Sci. Adv..

[124] Espadas I., Wingfield J.L., Nakahata Y., Chanda K., Grinman E., Ghosh I. (2024). Synaptically-targeted long non-coding RNA SLAMR promotes structural plasticity by increasing translation and CaMKII activity. Nat. Commun..

[125] Han X., Xu J., Chen Z., Li P., Zhao L., Tao J. (2022). Gas5 inhibition promotes the axon regeneration in the adult mammalian nervous system. Exp. Neurol..

[126] Swarnkar S., Avchalumov Y., Espadas I., Grinman E., Liu X.A., Raveendra B.L. (2021). Molecular motor protein KIF5C mediates structural plasticity and long-term memory by constraining local translation. Cell Rep..

[127] Li D., Zhang J., Wang M., Li X., Gong H., Tang H. (2018). Activity dependent LoNA regulates translation by coordinating rRNA transcription and methylation. Nat. Commun..

[128] Jankowsky E., Harris M.E. (2015). Specificity and nonspecificity in RNA-protein interactions. Nat. Rev. Mol. Cell Biol..

[129] Dominguez D., Freese P., Alexis M.S., Su A., Hochman M., Palden T. (2018). Sequence, structure, and context preferences of human RNA binding proteins. Mol. Cell.

[130] Van Nostrand E.L., Freese P., Pratt G.A., Wang X., Wei X., Xiao R. (2020). A large-scale binding and functional map of human RNA-binding proteins. Nature.

[131] Rot G., Wang Z., Huppertz I., Modic M., Lenče T., Hallegger M. (2017). High-resolution RNA maps suggest common principles of splicing and polyadenylation regulation by TDP-43. Cell Rep..

[132] Stefl R., Skrisovska L., Allain F.H. (2005). RNA sequence- and shape-dependent recognition by proteins in the ribonucleoprotein particle. EMBO Rep..

[133] Lambert N., Robertson A., Jangi M., McGeary S., Sharp P.A., Burge C.B. (2014). RNA Bind-n-Seq: quantitative assessment of the sequence and structural binding specificity of RNA binding proteins. Mol. Cell.

[134] Sanchez de Groot N., Armaos A., Graña-Montes R., Alriquet M., Calloni G., Vabulas R.M. (2019). RNA structure drives interaction with proteins. Nat. Commun..

[135] Jolma A., Zhang J., Mondragón E., Morgunova E., Kivioja T., Laverty K.U. (2020). Binding specificities of human RNA-binding proteins toward structured and linear RNA sequences. Genome Res..

[136] Chen X., Mayr C. (2022). A working model for condensate RNA-binding proteins as matchmakers for protein complex assembly. RNA.

[137] Kishor A., White E.J.F., Matsangos A.E., Yan Z., Tandukar B., Wilson G.M. (2017). Hsp70's RNA-binding and mRNA-stabilizing activities are independent of its protein chaperone functions. J. Biol. Chem..

[138] Khvorova A., Kwak Y.G., Tamkun M., Majerfeld I., Yarus M. (1999). RNAs that bind and change the permeability of phospholipid membranes. Proc. Natl. Acad. Sci. U. S. A..

[139] Vlassov A., Khvorova A., Yarus M. (2001). Binding and disruption of phospholipid bilayers by supramolecular RNA complexes. Proc. Natl. Acad. Sci. U. S. A..

[140] Czerniak T., Saenz J.P. (2022). Lipid membranes modulate the activity of RNA through sequence-dependent interactions. Proc. Natl. Acad. Sci. U. S. A..

[141] Wang X., Han X., Powell C.A. (2022). Lipids and genes: regulatory roles of lipids in RNA expression. Clin. Transl Med..

[142] Donia T., Jyoti B., Suizu F., Hirata N., Tanaka T., Ishigaki S. (2019). Identification of RNA aptamer which specifically interacts with PtdIns(3)P. Biochem. Biophys. Res. Commun..

[143] Bayona-Hernandez A., Guerra S., Jiménez-Ramirez I.A., Sztacho M., Hozak P., Rodriguez-Zapata L.C. (2023). LIPRNAseq: a method to discover lipid interacting RNAs by sequencing. Mol. Biol. Rep..

[144] Suga K., Umakoshi H., Tomita H., Tanabe T., Shimanouchi T., Kuboi R. (2010). Liposomes destabilize tRNA during heat stress. Biotechnol. J..

[145] Marty R., N'soukpoé-Kossi C.N., Charbonneau D.M., Kreplak L., Tajmir-Riahi H.A. (2009). Structural characterization of cationic lipid-tRNA complexes. Nucleic Acids Res..

[146] Lin A., Hu Q., Li C., Xing Z., Ma G., Wang C. (2017). The LINK-A lncRNA interacts with PtdIns(3,4,5)P. Nat. Cell Biol..

[147] Li R.H., Tian T., Ge Q.W., He X.Y., Shi C.Y., Li J.H. (2021). A phosphatidic acid-binding lncRNA SNHG9 facilitates LATS1 liquid-liquid phase separation to promote oncogenic YAP signaling. Cell Res..

[148] Han L., Huang D., Wu S., Liu S., Wang C., Sheng Y. (2023). Lipid droplet-associated lncRNA LIPTER preserves cardiac lipid metabolism. Nat. Cell Biol..

[149] Licatalosi D.D., Mele A., Fak J.J., Ule J., Kayikci M., Chi S.W. (2008). HITS-CLIP yields genome-wide insights into brain alternative RNA processing. Nature.

[150] Huppertz I., Attig J., D'Ambrogio A., Easton L.E., Sibley C.R., Sugimoto Y. (2014). iCLIP: protein-RNA interactions at nucleotide resolution. Methods.

[151] Danan C., Manickavel S., Hafner M. (2016). PAR-CLIP: a method for transcriptome-wide identification of RNA binding protein interaction sites. Methods. Mol. Biol..

[152] Sayad A., Badrlou E., Ghafouri-Fard S., Taheri M. (2020). Association analysis between the rs1899663 polymorphism of HOTAIR and risk of psychiatric conditions in an Iranian population. J. Mol. Neurosci..

[153] Hafner M., Landthaler M., Burger L., Khorshid M., Hausser J., Berninger P. (2010). Transcriptome-wide identification of RNA-binding protein and microRNA target sites by PAR-CLIP. Cell.

[154] Chanda K., Grinman E., Clark K., Sadhu A., Raveendra B., Swarnkar S. (2025). The lncRNA Gas5 is an activity-responsive scaffold that mediates cAMP-dependent synaptic plasticity. Sci. Signal..

[155] Chen M., Manley J.L. (2009). Mechanisms of alternative splicing regulation: insights from molecular and genomics approaches. Nat. Rev. Mol. Cell Biol..

[156] Wang Y., Liu J., Huang B.O., Xu Y.M., Li J., Huang L.F. (2015). Mechanism of alternative splicing and its regulation. Biomed. Rep..

[157] Lamond A.I. (1993). The spliceosome. Bioessays.

[158] Will C.L., Lührmann R. (2011). Spliceosome structure and function. Cold Spring Harb Perspect. Biol..

[159] Romero-Barrios N., Legascue M.F., Benhamed M., Ariel F., Crespi M. (2018). Splicing regulation by long noncoding RNAs. Nucleic Acids Res..

[160] Malakar P., Shukla S., Mondal M., Kar R.K., Siddiqui J.A. (2024). The nexus of long noncoding RNAs, splicing factors, alternative splicing and their modulations. RNA Biol..

[161] Marima R., Francies F.Z., Hull R., Molefi T., Oyomno M., Khanyile R. (2021). MicroRNA and alternative mRNA splicing events in cancer drug response/resistance: potent therapeutic targets. Biomedicines.

[162] Yap K., Mukhina S., Zhang G., Tan J.S.C., Ong H.S., Makeyev E.V. (2018). A short tandem repeat-enriched RNA assembles a nuclear compartment to control alternative splicing and promote cell survival. Mol. Cell.

[163] Tian B., Manley J.L. (2017). Alternative polyadenylation of mRNA precursors. Nat. Rev. Mol. Cell Biol..

[164] Mayr C. (2019). What are 3' UTRs doing?. Cold Spring Harb Perspect. Biol..

[165] Tushev G., Glock C., Heumüller M., Biever A., Jovanovic M., Schuman E.M. (2018). Alternative 3' UTRs modify the localization, regulatory potential, stability, and plasticity of mRNAs in neuronal compartments. Neuron.

[166] Middleton S.A., Eberwine J., Kim J. (2019). Comprehensive catalog of dendritically localized mRNA isoforms from sub-cellular sequencing of single mouse neurons. BMC Biol..

[167] Zheng D., Wang R., Ding Q., Wang T., Xie B., Wei L. (2018). Cellular stress alters 3'UTR landscape through alternative polyadenylation and isoform-specific degradation. Nat. Commun..

[168] Yang X., Hu X., Liu J., Wang R., Zhang C., Han F. (2020). N6-methyladenine modification in noncoding RNAs and its function in cancer. Biomark Res..

[169] Tang P., Yang J., Chen Z., Du C., Yang Y., Zhao H. (2024). Nuclear retention coupled with sequential polyadenylation dictates post-transcriptional m^6^A modification in the nucleus. Mol. Cell.

[170] Shi Z., Wen K., Zou Z., Fu W., Guo K., Sammudin N.H. (2024). YTHDF1 mediates translational control by m^6^A mRNA methylation in adaptation to environmental challenges. bioRxiv.

[171] Mitsuhashi H., Nagy C. (2023). Potential roles of m^6^A and FTO in synaptic connectivity and major depressive disorder. Int. J. Mol. Sci..

[172] Li J., Wang X., Wang H. (2024). RNA modifications in long non-coding RNAs and their implications in cancer biology. Bioorg. Med. Chem..

[173] Zhou K.I., Pecot C.V., Holley C.L. (2024). 2'-. RNA.

[174] Lv X., Zhang R., Li S., Jin X. (2024). tRNA modifications and dysregulation: implications for brain diseases. Brain. Sci..

[175] Blaze J., Akbarian S. (2022). The tRNA regulome in neurodevelopmental and neuropsychiatric disease. Mol. Psychiatry.

[176] Kapur M., Molumby M.J., Guzman C., Heinz S., Ackerman S.L. (2024). Cell-type-specific expression of tRNAs in the brain regulates cellular homeostasis. Neuron.

[177] Flynn R.A., Pedram K., Malaker S.A., Batista P.J., Smith B.A.H., Johnson A.G. (2021). Small RNAs are modified with N-glycans and displayed on the surface of living cells. Cell.

[178] Safari M., Noroozi R., Taheri M., Ghafouri-Fard S. (2020). The rs12826786 in HOTAIR lncRNA is associated with risk of autism spectrum disorder. J. Mol. Neurosci..

[179] Kerin T., Ramanathan A., Rivas K., Grepo N., Coetzee G.A., Campbell D.B. (2012). A noncoding RNA antisense to moesin at 5p14.1 in autism. Sci. Transl Med..

[180] Rao S.Q., Hu H.L., Ye N., Shen Y., Xu Q. (2015). Genetic variants in long non-coding RNA MIAT contribute to risk of paranoid schizophrenia in a Chinese Han population. Schizophr Res..

[181] Bilinovich S.M., Lewis K., Grepo N., Campbell D.B. (2019). The long noncoding RNA. Front Genet..

[182] Wang H., Wu X., Chen Y., Hou F., Zhu K., Jiang Q. (2023). Combining multi-omics approaches to prioritize the variant-regulated functional long non-coding RNAs in autism spectrum disorder. Asian J. Psychiatr..

[183] Ye N., Rao S., Du T., Hu H., Liu Z., Shen Y. (2017). Intergenic variants may predispose to major depression disorder through regulation of long non-coding RNA expression. Gene.

[184] Liu W., Li W., Cai X., Yang Z., Li H., Su X. (2020). Identification of a functional human-unique 351-bp Alu insertion polymorphism associated with major depressive disorder in the 1p31.1 GWAS risk loci. Neuropsychopharmacology.

[185] Namvar A., Kahaei M.S., Fallah H., Nicknafs F., Ghafouri-Fard S., Taheri M. (2020). ANRIL variants are associated with risk of neuropsychiatric conditions. J. Mol. Neurosci..

[186] Márki S., Göblös A., Szlávicz E., Török N., Balicza P., Bereznai B. (2018). The rs13388259 intergenic polymorphism in the genomic context of the. Parkinsons Dis..

[187] Shadkam R., Saadat P., Azadmehr A., Chehrazi M., Daraei A. (2024). Key non-coding variants in three neuroapoptosis and neuroinflammation-related lncRNAs are protectively associated with susceptibility to Parkinson’s disease and some of its clinical features. Mol. Neurobiol..

[188] Eftekharian M., Noroozi R., Komaki A., Mazdeh M., Taheri M., Ghafouri-Fard S. (2019). GAS5 genomic variants and risk of multiple sclerosis. Neurosci. Lett..

[189] Moradi M., Gharesouran J., Ghafouri-Fard S., Noroozi R., Talebian S., Taheri M. (2020). Role of NRC1 and GAS5 gene polymorphisms in multiple sclerosis. Int. J. Neurosci..

[190] Eftekharian M.M., Noroozi R., Komaki A., Mazdeh M., Ghafouri-Fard S., Taheri M. (2019). MALAT1 genomic variants and risk of multiple sclerosis. Immunol. Invest.

[191] Sattari F.N., Rezvan N. (2020). The role of single nucleotide polymorphisms within long non-coding RNAs in susceptibility to human disorders. Ecol. Genet. Genomics.

[192] Taheri M., Noroozi R., Sadeghpour S., Omrani M.D., Ghafouri-Fard S. (2020). The rs4759314 SNP within Hotair lncRNA is associated with risk of multiple sclerosis. Mult. Scler. Relat. Disord..

[193] Bahrami T., Taheri M., Omrani M.D., Karimipoor M. (2020). Associations between genomic variants in lncRNA-TRPM2-AS and lncRNA-HNF1A-AS1 genes and risk of multiple sclerosis. J. Mol. Neurosci..

[194] Chen G., Qiu C., Zhang Q., Liu B., Cui Q. (2013). Genome-wide analysis of human SNPs at long intergenic noncoding RNAs. Hum. Mutat..

[195] Policarpo R., d'Ydewalle C. (2021). Missing *lnc*(RNAs) in alzheimer’s disease?. Genes (Basel).

[196] Lambert J.C., Ibrahim-Verbaas C.A., Harold D., Naj A.C., Sims R., Bellenguez C. (2013). Meta-analysis of 74,046 individuals identifies 11 new susceptibility loci for Alzheimer's disease. Nat. Genet..

[197] de Rojas I., Moreno-Grau S., Tesi N., Grenier-Boley B., Andrade V., Jansen I.E. (2021). Common variants in Alzheimer's disease and risk stratification by polygenic risk scores. Nat. Commun..

[198] Wingo T.S., Liu Y., Gerasimov E.S., Vattathil S.M., Wynne M.E., Liu J. (2022). Shared mechanisms across the major psychiatric and neurodegenerative diseases. Nat. Commun..

[199] Zhu X., Need A.C., Petrovski S., Goldstein D.B. (2014). One gene, many neuropsychiatric disorders: lessons from Mendelian diseases. Nat. Neurosci..

[200] Gratten J., Visscher P.M., Mowry B.J., Wray N.R. (2013). Interpreting the role of de novo protein-coding mutations in neuropsychiatric disease. Nat. Genet..

[201] Banerjee D., Sultana S., Banerjee S. (2024). Gas5 regulates early-life stress-induced anxiety and spatial memory. J. Neurochem..

[202] Martens L., Rühle F., Witten A., Meder B., Katus H.A., Arbustini E. (2021). A genetic variant alters the secondary structure of the lncRNA H19 and is associated with dilated cardiomyopathy. RNA Biol..

[203] Chen B.Y., Lin J.J., Lu M.K., Tan H.P., Jang F.L., Lin S.H. (2021). Neurodevelopment regulators miR-137 and miR-34 family as biomarkers for early and adult onset schizophrenia. NPJ Schizophr.

[204] Yin J., Lin J., Luo X., Chen Y., Li Z., Ma G. (2014). miR-137: a new player in schizophrenia. Int. J. Mol. Sci..

[205] Guan F., Zhang B., Yan T., Li L., Liu F., Li T. (2014). MIR137 gene and target gene CACNA1C of miR-137 contribute to schizophrenia susceptibility in Han Chinese. Schizophr. Res..

[206] Siegert S., Seo J., Kwon E.J., Rudenko A., Cho S., Wang W. (2015). The schizophrenia risk gene product miR-137 alters presynaptic plasticity. Nat. Neurosci..

[207] Mokhtari M., Sargazi S., Saravani R., Heidari Nia M., Mirinejad S., Hadzsiev K. (2022). Genetic polymorphisms in miR-137 and its target genes, TCF4 and CACNA1C, contribute to the risk of bipolar disorder: a preliminary case-control Study and bioinformatics analysis, disease markers. Dis. Markers..

[208] Mahmoudi E., Cairns M.J. (2017). MiR-137: an important player in neural development and neoplastic transformation. Mol. Psychiatry.

[209] Werner A., Kanhere A., Wahlestedt C., Mattick J.S. (2024). Natural antisense transcripts as versatile regulators of gene expression. Nat. Rev. Genet..

[210] Modarresi F., Pedram Fatemi R., Razavipour S.F., Ricciardi N., Makhmutova M., Khoury N. (2021). A novel knockout mouse model of the noncoding antisense. Heliyon.

[211] McCartan R., Khorkova O., Volmar C.H., Wahlestedt C. (2023). Nucleic acid-based therapeutics for the treatment of central nervous system disorders. Front. Genet..

